# Accumulation of Abnormal Amyloplasts in Pulp Cells Induces Bitter Pit in *Malus domestica*

**DOI:** 10.3389/fpls.2021.738726

**Published:** 2021-09-23

**Authors:** Lina Qiu, Shanshan Hu, Yongzhang Wang, Haiyong Qu

**Affiliations:** College of Horticulture, Qingdao Agricultural University, Qingdao, China

**Keywords:** programmed cell death, vacuole, calcium, domestic apple, mitochondria, transmission electron microscope

## Abstract

Apple bitter pit primarily occurs during fruit ripening and storage; however, its formation mechanism remains unclear. Although it is considered that Ca^2+^ deficiency causes metabolic disorders in apples, there have been few studies on the mechanism of the bitter pit from the perspective of cell structure. At the fruit ripening stage, the fruit with a bitter pit on the tree was taken as the research material. In this study, the microscopic observation revealed numerous amyloplasts in the pulp cells of apples affected with bitter pit, but not in the healthy pulp. Furthermore, the results of fluorescence staining and transmission electron microscopy (TEM) revealed that the bitter pit pulp cells undergo programmed cell death (PCD), their nuclear chromosomes condense, and amyloplast forms autophagy. The cytoplasmic Ca^2+^ concentration in the healthy fruits was lowest near the peduncle, followed by that in the calyx, whereas it was highest at the equator. In contrast, the cytoplasmic Ca^2+^ concentration in apple fruits showing bitter pit disorder was lowest near the peduncle and highest in the calyx. Moreover, the cytosolic Ca^2+^ concentration in the flesh cells of apples with the bitter pit was much lower than that in the healthy apple flesh cells; however, the concentration of Ca^2+^ in the vacuoles of fruits with the bitter pit was higher than that in the vacuoles of healthy fruits. In summary, bitter pit pulp cells contain a large number of amyloplasts, which disrupts the distribution of Ca^2+^ in the pulp cells and causes PCD. These two processes lead to an imbalance in cell metabolism and induce the formation of a bitter pit.

## Introduction

An apple (*Malus domestica*), belonging to the family Rosaceae, is a deciduous tree with the second largest global harvest area after citrus. However, an apple bitter pit is a major physiological disorder that affects the economic characteristics of apples worldwide. For example, in 2016, 74% of “Honeycrisp” apples in orchards of Pennsylvania (American) developed bitter pit (Baugher et al., 2017). Apple bitter pit should be referred to as a disorder rather than a disease (Parbery, 2015), as it is not associated with fungi, bacteria, or viruses. Bitter pit involves complicated developmental processes (Ferguson and Watkins, 1989). It has been the most studied apple disorder since its discovery over a century ago. However, its formation mechanism has not yet been thoroughly studied. There is a significant correlation between Ca^2+^ concentration and fruit quality, as Ca^2+^ regulates fruit firmness, color, and soluble solid content, among other characteristics. There are 35 types of physiological disorders related to Ca^2+^ concentration in fruits (Freitas and Mitcham, 2012). Several studies have shown that the incidence of a bitter pit is primarily related to the absorption and distribution of calcium in fruits (Neilsen et al., 2005; Sharma et al., 2014), which mainly occurs near the calyx of the apple. During agricultural production, foliar calcium spraying, soil calcium application, and fruit postharvest calcium soaking are employed to reduce the incidence of bitter pit (Blanco et al., 2010; Falchi et al., 2017); however, the incidence of bitter pit in apple fruit has not yet been effectively controlled. For instance, Ernani et al. (2008) carried out spraying experiments on the apples for several years in succession. The period of calcium spraying included the time from flowering to 1 week before harvest, but the incidence of bitter pit remained extremely high (Ferguson and Watkins, 1989; Ernani et al., 2008). The results from various studies on the regulation of Ca^2+^ in the apples with bitter pit are contradictory (Ernani et al., 2008). Although some researchers believe that bitter pit is positively correlated with high [K + Mg]/Ca, Mg/Ca, and K/Ca ratios (do Amarante et al., 2013; Miqueloto et al., 2014; Liu et al., 2021), changes occurring in the structure of flesh cells of apples with bitter pit remain largely unknown, and the role of Ca^2+^ in the formation of bitter pit remains unclear (Torres et al., 2015).

Wilson and Bacic (2012) suggested that researchers should return to the cellular level and study the mechanisms of cellular behavior. Based on the ultrastructure of the cells, changes in the plasmids of three different types of apple pulp cells were analyzed. It is important to understand the complex biological changes in plasmid functions (Schaeffer et al., 2017). The distribution of Ca^2+^ in various cell compartments is affected by various types of stimulation (Meldolesi and Grohovaz, 2001). In a previous study, the ultra-microscopic observation of apple apoplasmic phloem revealed the unloading mechanism of sorbitol during apple development (Zhang et al., 2004). The apple was treated with Ca(NO_3_)_2_, CaCl_2_, and Ca chelated with EDTA, and the structure of epidermal and hypodermal cells of the fruit was observed ultramicroscopically. Ca^2+^ contributes to the stability of cell structure (Kowalik et al., 2020). The distribution of Ca^2+^ in various cell compartments is affected by various types of stimulation; however, these changes can only be observed at the ultrastructural level (Sedmíková et al., 2003; Kowalik et al., 2020). In another study, the ultramicroscopic analysis of the pulp cells of apples with bitter pit revealed plasmolysis and cell membrane rupture (de Freitas et al., 2010); however, the type and mechanism of cell death remain unclear.

Currently, the “Fuji” apple is an economically important apple cultivar worldwide and is commercially grown in Japan, China, United States, Australia, and South Africa (Chigwaya et al., 2021). In China, ‘Fuji’ is the main planting variety, and its yield and cultivated area account for more than 70% of the total apple production and total cultivated area (Zhang et al., 2018). However, “Fuji” apples are prone to bitter pit (do Amarante et al., 2013). This study aimed to analyze the microstructure of pulp cells and the distribution of calcium ions in “Fuji” apples with bitter pit, and explore the formation mechanism of apple bitter pit.

## Materials and Methods

### Materials

“Fuji” apples were obtained from the Laixi seedling breeding farm (Qingdao city, China, 120°28E, 36°51N). During the ripening season in autumn, the fruits suffering from the bitter pit were selected and removed from the tree. Healthy and undamaged fruits were used as controls.

### Methods

#### Cell Microscopic Observation

The bitter pit-affected and healthy fruits were washed and dried; then, the bitter pit and healthy tissues were sectioned by hand slicing under a stereo microscope, and structural differences were observed under an optical microscope (Model: DM2500, LEICA, Germany).

#### Annexin V-FITC/PI Dying

Apoptotic cells were assessed using the Annexin V-FITC Detection Kit (Vazyme, China) according to the protocols of the manufacturer. After pre-cooling, 2–3 mm of flesh under the exocarp was cut into 2 mm × 2 mm × 3 mm cuboids on ice, embedded in optimal cutting temperature (OCT), and placed on a quick-freezing rack. A 25 μm-thick sheet was cut using a freezing microtome (Model: HM525, LEICA, Germany). The cells were washed two times with cold PBS and suspended in the binding buffer. The samples were stained with 10 μl of Annexin V-FITC and 10 μl of PI for 20 min at room temperature (25°C) in the dark. The apoptotic index was immediately determined using a confocal laser scanning microscope (TCS SP5 II, Leica, Germany).

#### Ultrastructural Observation

The fixation and embedding of apple pulp samples refer to the methods of de Freitas et al. (2010), which are slightly changed according to the characteristics of the samples. Approximately, 2–3 mm of flesh under the exocarp was cut into 1 mm × 1 mm × 2 mm cuboids. The cuboids were fixed with 3% glutaraldehyde in 10 mmol/L PBS (pH 7.2) for 2 h at room temperature, then embedded in 2% agar, and fixed in fresh fixatives under vacuum for 3 h at 4°C. After rinsing with distilled water, the sections were incubated in a 0.5% aqueous uranyl acetate solution at room temperature for 2 h. After another two washes with distilled water, the plant material was dehydrated in a series of acetone at successive concentrations of 30% (10 min), 50% (10 min), 70% (10 min), 90% (10 min), 95% (30 min), and 100% (30 min, two times). The dehydrated plant samples were embedded in Eponate 12 resin (Ted Pella Inc., Redding, CA, United States) and polymerized at 45°C for 12 h and 60°C for 48 h. The fixed and dehydrated pulp cells were prepared for TEM observation. Ultrathin sections (60 nm) were cut with an ultramicrotome (Model: UC7, Leica, Germany) and stained with uranyl acetate/lead citrate. The sections were examined using a Hitachi TEM system (Model: HT7700) at 80 kV.

The potassium pyroantimonate method was used to determine free Ca^2+^ in the cells. The fixation and embedding of apple pulp samples were performed as described by de Freitas et al. (2010). To verify that the black particles observed *via* electron microscopy were Ca^2+^, the ultrathin slices of the pulp tissue with bitter pit disorder were chelated with 0.2 mol/L EGTA (Qin et al., 2005).

#### Nuclear 4′,6-Diamidino-2-Phenylindole Staining

First, 2 g of the sample was weighed and ground to a powder using liquid nitrogen; then, nuclei were extracted using the Plant Cell Nuclear Extraction Kit (Product Number: NXTRACT, Sigma-Aldrich, MO, United States) according to the instruction of the manufacturer. The extracted nuclei were stained with DAPI and observed under a fluorescence microscope (EVOS FL Auto 2, Thermo Fisher Scientific, MA, United States).

#### Optical Microscope Observation of Amyloplasts

To observe the amyloplasts in bitter pit cells, the pulp was soaked in a fixative (5% (v/v) formaldehyde, 5% (v/v) acetic acid, and 45% (v/v) ethanol) at 4°C for 48 h. The fixed pulp was stained with an I_2_-KI solution (0.15% (w/v) I_2_ and 0.45% (w/v) KI) for 5 min. Then, the fruit pulp was observed under a light microscope (EVOS Auto 2, Thermo Fisher Scientific, MA, United States) (Takahashi et al., 2003).

#### Apoplastic Water-Soluble Ca^2+^ Determination

The extraction and determination of water-soluble mineral elements in the apoplast were conducted according to the methods described by de Freitas et al. (2012). Briefly, the outer skin of the healthy and bitter pit fruits was removed with a stainless-steel knife, pulp with a diameter of 1.5 cm was retrieved with a punch perpendicular to the projection position, about 3 mm of the flesh of the surface layer was cut, and a pulp disc with a diameter of 1.5 cm, the thickness of about 3 mm, and weight of 0.40–0.45 g was obtained. The pulp disc was added to ddH_2_O for 10 s, the water was sucked up with paper, and the disc was placed into a funnel to extract the mineral elements of the apoplasts; finally, the Whatman filter paper was placed into the funnel and used for filtering 200 μl isotonic mannitol (0.31 mol/L). The funnel samples were eluted with 700 μl of isotonic mannitol under a 25 mm Hg vacuum (70 μl each time). Three discs were obtained from three fruits each, providing a total of nine pulp discs. The extraction liquid volume of each sample was approximately 6.3 ml. The apoplastic Ca^2+^ content was measured using inductively coupled plasma mass spectroscopy (Agilent 7700, Agilent Technologies Inc., Santa Clara, CA, United States).

#### Transcriptome Analysis

The calyx end pulp of healthy fruit, the calyx end healthy pulp of bitter pit fruit, and the pulp of bitter pit were selected for the transcriptomic analysis. RNAs from the samples were extracted using TRIzol Reagent (Life Technologies, Carlsbad, CA, United States) in three biological replicates as per the instructions from the manufacturer. RNA quality and concentration were verified using the Agilent 2100 Bioanalyzer (Agilent Technologies, Inc., Santa Clara, CA, United States). RNA reverse transcription into cDNA (TIANGEN Biotech, Beijing Co., Ltd, China). The transcribed cDNA was used to construct cDNA libraries using the NEBNext Ultra RNA Library Prep Kit from Illumina (NEB, E7530, MA, United States). The cDNA library was sequenced on a paired-end (PE) flow cell using Illumina HiSeq 2500 sequencing platform (Illumina Inc., San Diego, CA, United States). Beijing Biomarker Technologies^1^ provided the commercially available experimental procedures. The transcriptomic data that support the findings of this article are accessible under NCBI’s BioProject with accession number PRJNA733599 and SRA accession numbers SRR14684876, SRR14684877, and SRR14684878.

#### Fluo-4/AM Staining

The fruits were consistently light, and the fruit size was uniform. The apples were cut into four parts on average, and each portion of pulp was taken from near the peduncle to near the calyx. Seven healthy apples and six apples showing bitter pit symptoms were used to isolate the protoplasts from the pulp cells, which were then fluorescently stained with fluo-4/AM (Qiu et al., 2020). The final concentration of fluo-4/AM (Dojindo Laboratories, Kumamoto, Japan) was 5 μmol/L. Since the excitation wavelength of fluo-4/AM is 490 nm, GFP was selected as the light cube. The viability of cells was then determined under a fluorescent microscope (EVOS Auto 2, Thermo Fisher Scientific, United States). The fluorescence results were analyzed using Image-Pro Plus 6.0 software (Media Cybernetics, Inc., MD, United States), according to our published methods (Qu et al., 2016).

#### Transformation by *in vivo* Fruit Injection

The target gene was transformed into Agrobacterium and propagated in LB medium, then centrifuged, collected sedimentation, and put into MES medium for activation. Immature tomatoes (green ripened) were selected for *in vivo* injection. Injection methods refer to Yasmeen et al. (2009). All tomatoes were injected two times on two consecutive days. Ten fruits were used for each treatment, and both experiments were carried out three times. Fruits injected with agrobacterium without target gene were used as a negative control. The tomatoes were harvested a week after injection.

### Statistical Analyses

Microsoft Office 365 was used for data processing. Statistical analysis was performed using GraphPad Prism 7.0 software (GraphPad Software, Inc., La Jolla, CA, United States). The student’s *t*-test was used to analyze the differences among the experimental groups.

## Results

### Microscopic Observation of the Pulp Cells of Apples With Bitter Pit

**FIGURE 1:**
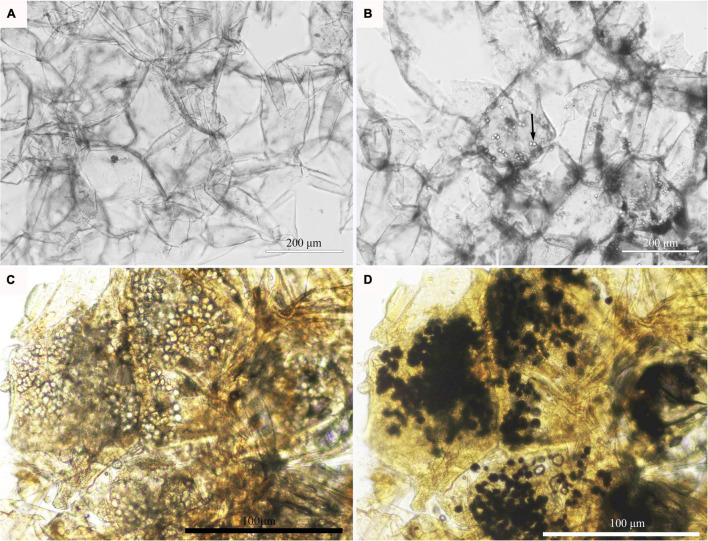
Microscopic observation of amyloplasts in the flesh cells of apples with bitter pit. **(A)** The flesh of healthy fruit. **(B)** The bitter pit flesh; the cells contain granular substances. **(C)** Pulp cells from bitter pit-affected fruits without I_2_-IK staining. **(D)** Pulp cells from bitter pit-affected fruits stained with I-IK. Granular material is shown in black and purple. The magnification of the objective of the microscope is 20 times.

**FIGURE 2:**
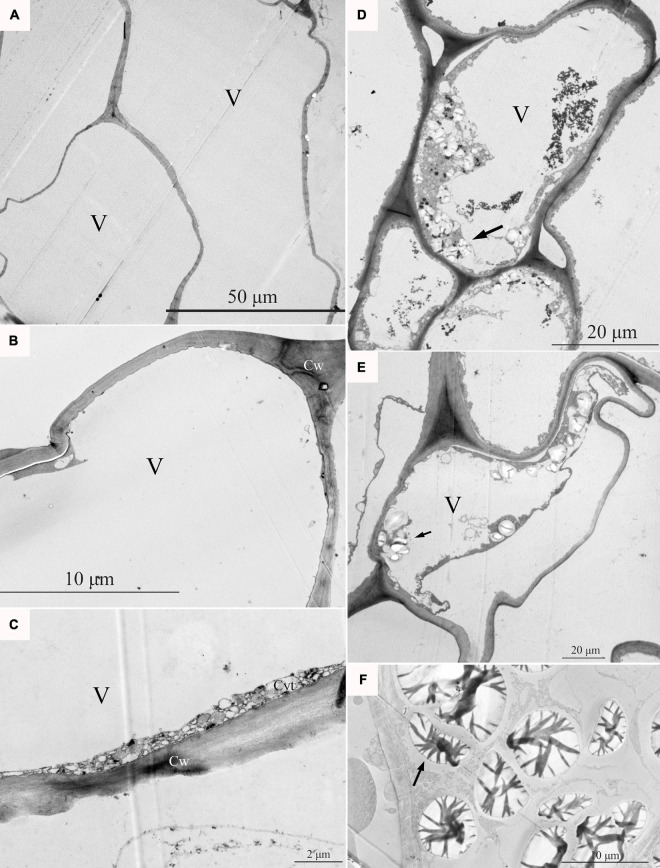
The pulp cells were observed *via* transmission electron microscopy (TEM). **(A–C)** Pulp cells of a healthy fruit; healthy pulp cells gradually enlarge. **(D)** The ultramicroscopic observation of amyloplasts in bitter pit flesh cells. **(E)** Analysis of the flesh cells of bitter pit in apples revealed plasmolysis and abundant amyloplasts. **(F)** Amyloplasts further enlarged. Black arrows indicate amyloplasts. V, vacuole; Cw, cell wall.

At the fruit ripening stage, the apples growing on the tree were affected with bitter pit (Supplementary Figure 1A). The symptoms included small dark depressions near the calyx end of the fruit caused by the collapse of flesh cells just below the peel (Supplementary Figures 1B–D). After sections of the fruit were picked from the tree, no organelles were observed in the healthy pulp cells *via* optical microscopy (Figure 1A). However, there was abundant granular material in the pulp cells of apples with bitter pit (Figure 1B). This granular material showed black and purple color after I_2_-IK staining (Figures 1C,D), indicating that it contained starch. Upon observing the healthy fruit cells *via* transmission electron microscopy (TEM), the cell membrane surface was found to be smooth without protrusions, and the vacuole had no obvious inclusion and did not undergo plasmolysis (Figures 2A–C). However, the protoplasts of the flesh cells of apples with bitter pit extended into vacuoles (Figures 2D,E), showed plasmolysis (Figures 2D,E), and contained numerous amyloplasts (Figures 2D–F). These observations indicated the accumulation of amyloplasts in the flesh cells of apples with bitter pit.

### Ultrastructural Detection of Intracellular Free Calcium

**FIGURE 3:**
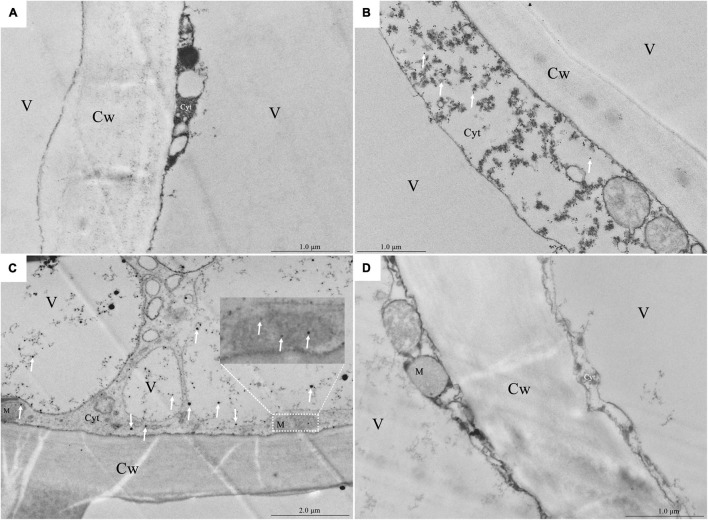
Localization of calcium in the pulp cells *via* potassium pyroantimonate precipitation. **(A)** Healthy fruit pulp cell fragments. **(B)** Pulp cell fragments of the healthy part of the fruit with bitter pit taken from an area close to the bitter pit spot part. **(C)** Fragments of pulp cells with bitter pit. **(D)** The ultrathin section of the fragments of pulp cells from apples with the bitter pit was treated with 0.2 mol/L EGTA. The white arrow points to Ca^2+^ precipitation. Cw, cell wall; M, mitochondria; Cyt, cytoplasm; V, vacuole.

As the vacuole occupied the center of the flesh cell of the healthy fruit and the cytoplasm was squeezed around the cell, it was difficult to observe Ca^2+^ precipitation (Figure 3A). In the fruit with a bitter pit, the healthy pulp cells near the bitter pit location showed plasmolysis. Extensive Ca^2+^ precipitation was observed in the cytoplasm, but not in the vacuole (Figure 3B). The pulp cells of apples with bitter pit showed Ca^2+^ precipitation not only in the cytoplasm but also in the vacuole and mitochondria (Figure 3C). These results indicate that the concentration of free Ca^2+^ in the vacuoles of fruits with the bitter pit was higher than that of healthy pulp cells. No black precipitate was observed in the vacuoles of the ultrathin slices of the fruit with bitter pit treated with 0.2 mol/L EGTA, which indicated that the black precipitate observed was due to the precipitation of free Ca^2+^ (Figure 3D). In addition, the granular precipitation of Ca^2+^ on the tonoplast of the pulp cells of apples with bitter pit (Supplementary Figures 2A,B) was similar to that in the bean hypocotyl cells (*Phaseolus vulgaris* L) (Hanchey, 1982) and the shape of the Ca^2+^ flocculent precipitates (Supplementary Figures 2C,D) in the vacuoles were similar to that in rice (*Oryza sativa* L.) lodicules (Qin et al., 2005). The pulp cells from healthy and bitter pit parts of the fruit were observed under a microscope. No amyloplasts were observed in the healthy pulp cells of apples with bitter pit. Contrastingly, the pulp cells close to the bitter pit location contained small amounts of amyloplasts, while pulp cells from the bitter pit part contained numerous amyloplasts (Supplementary Figures 3A–C). This is consistent with the results of ultrastructural observations of the distribution of Ca^2+^ (Figures 3A–D). These findings suggest that the increase in Ca^2+^ concentration in the pulp cells is consistent with the accumulation of amyloplasts.

### Comparison of Apoplastic Water-Soluble Ca^2+^ Concentration and Distribution of Cytoplasmic Ca^2+^ in Fruits

**FIGURE 4:**
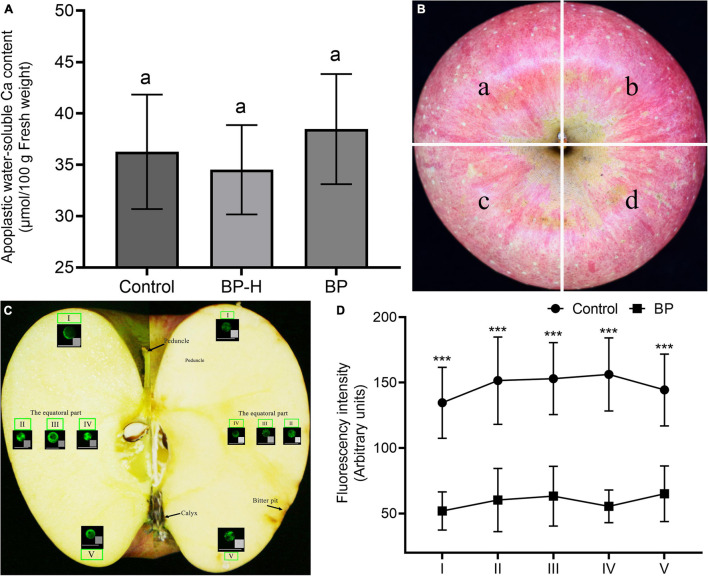
Apoplastic water soluble and cytoplasmic Ca^2+^. **(A)** The concentrations of water-soluble Ca^2+^ in different pulps near the calyx of fruit were compared. Data are represented as mean ± SE. The same letters indicate no significant difference (α = 0.05). Control, healthy fruit; BP-H, healthy pulp of bitter pit fruit; and BP, bitter pit fruit. **(B)** The image on the left shows a healthy fruit and that on the right shows a fruit with bitter pit disorder. The apple was divided into four sections on average. **(C)** Five pulp portions from each fruit section from near the peduncle to the calyx were sampled, and the pulp cell protoplasts were isolated, as shown in panels I, II, III, IV, and V. Fluorescent staining of cytoplasmic Ca^2+^ in apple fruit tissues from near the peduncle to near the calyx. Panels I–V show representative fluorescence images of protoplasts. **(D)** Ca^2+^ distribution in healthy fruit and bitter pit fruit. Seven healthy and bitter pit fruits were analyzed each. The number of healthy fruit protoplasts analyzed in each fruit section was as follows: I, *n* = 105; II, *n* = 92; III, *n* = 133; IV, *n* = 136; V, and *n* = 124. The number of bitter pit fruit protoplasts analyzed in each fruit section was as follows: I, *n* = 131; II, *n* = 142; III, *n* = 165; IV, *n* = 129; and V, *n* = 103. Values are presented as mean ± SE. ***Significant difference (*P* < 0.001). Control, healthy fruit; BP, bitter pit fruit. The magnification of the objective of the microscope is 20 times.

We measured the apoplastic water-soluble Ca^2+^ concentration at the calyx end of the fruit. The Ca^2+^ content of the fruit with the bitter pit was higher than that of healthy fruit, and the Ca^2+^ content of the healthy part of the fruit with the bitter pit was lower than that of healthy fruit, but the difference was not significant (Figure 4A). The fruit was divided into four parts (Figure 4B). Protoplasts were extracted from each part from the peduncle to the calyx and stained with fluo-4/AM (Figure 4C). In healthy fruit, the cytoplasmic Ca^2+^ concentration was lowest in the peduncle and calyx and highest in the equatorial part of the fruit. In the fruit with bitter pit, the cytoplasmic Ca^2+^ concentration was lowest in the peduncle end and inside the equator and highest in the calyx (Figure 4D). Thus, the distribution of cytoplasmic Ca^2+^ in fruits with the bitter pit was not consistent with that in the healthy fruits. Transcriptomic analysis revealed 431 common differential genes in the healthy fruit pulp cells, healthy pulp cells of fruits with bitter pit, and bitter pit pulp cells. Upon differential gene analysis using the BMKCloud platform^2^, six genes were found to be associated with Ca^2+^ concentration. The six genes were upregulated in the pulp of fruits with bitter pit (Supplementary Figure 4). This can explain why Ca^2+^ concentration in the pulp cells of fruits with the bitter pit was higher than that in other parts of the cells. In addition, the cytoplasmic Ca^2+^ concentration in each part of the pulp of healthy fruits was significantly (*P* < 0.001) higher than that in the corresponding portions of fruits with bitter pit symptoms (Figure 4D). These results suggest that cytoplasmic Ca^2+^ is associated with the occurrence of a bitter pit.

### Fluorescence-Based Detection of Programmed Death of Flesh Cells in Apples With Bitter Pit

**FIGURE 5:**
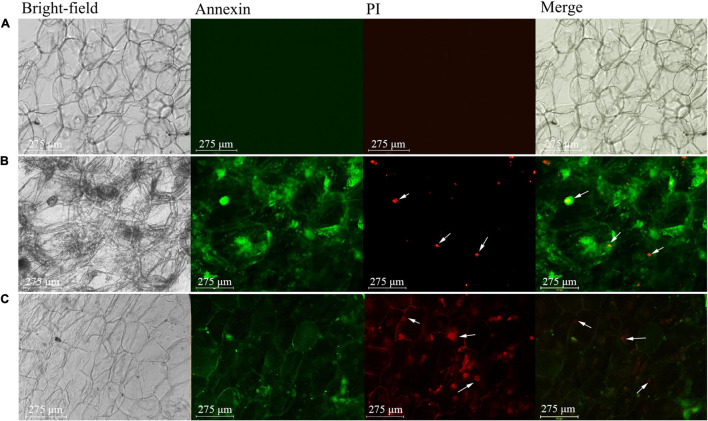
Fluorescence microscopic analysis of apple pulp cells. The cells were double-stained with Annexin V-FITC (green) and PI (red). **(A)** Healthy pulp cell. **(B)** Pulp cells of an apple with bitter pit. **(C)** Pulp cells of an apple treated with dexamethasone. The arrow indicates the nucleus. Bright field, optical photos without fluorescence. Annexin, green fluorescent channel. Green fluorescence indicates that the cell membrane could be stained. PI, red fluorescent channel. Red fluorescence indicates that the chromatin in the nucleus could be stained. Merge, the overlaying of the first three photos is used to illustrate the corresponding structure of the stained fluorescent cells. The magnification of the objective of the microscope is 20 times.

The results of Annexin V-FITC/PI staining revealed that the healthy flesh cells did not emit fluorescent signals in either green fluorescent protein (GFP) or red fluorescent protein (RFP) channels (Figure 5A). This indicated that the cell membranes of healthy and mature pulp cells were intact without losing the selection permeability. However, the pulp cells of apples with bitter pit emitted green fluorescence in the cell membrane in the GFP channel, and the cell nucleus emitted red fluorescence in the RFP channel (Figure 5B). This indicated that the cells exhibited apoptotic characteristics, such as phosphatidylserine eversion and loss of selective permeability of the cell membrane. When the cell membrane loses its selective permeability, propidium iodide (PI) can enter the cell and bind with chromatin in the nucleus to emit red fluorescence. We used dexamethasone, which is a programmed cell death (PCD) inducer, to treat flesh cells (Bonapace et al., 2010). The fluorescence staining results of dexamethasone-treated flesh cells were similar to those of flesh cells of apples with bitter pit (Figure 5C). These findings indicate that the flesh cells of apples with bitter pit undergo PCD.

### Nuclear Pathological Examination

**FIGURE 6:**
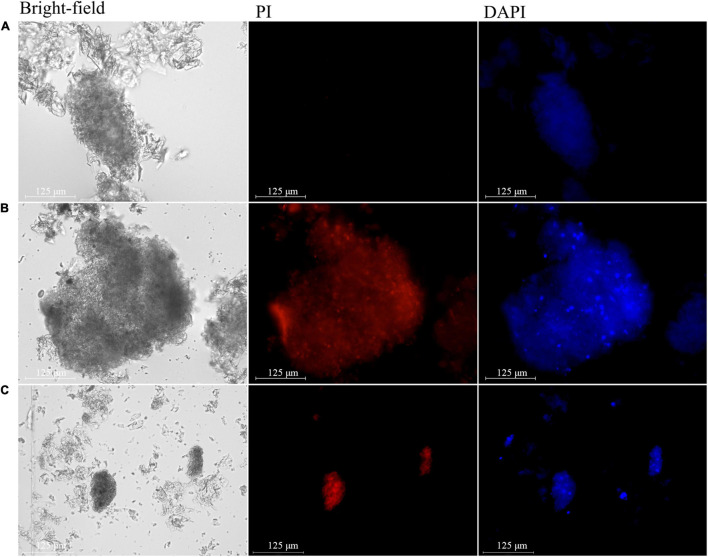
Changes in the nucleus during programmed cell death (PCD). **(A)** Nucleus extracted from healthy pulp cells. Propidium iodide (PI) and 4′,6-diamidino-2-phenylindole (DAPI) positively stained the nucleus. **(B)** Nucleus extracted from the flesh cells of apple with bitter pit. **(C)** Nucleus extracted from dexamethasone-treated flesh cells. The magnification of the objective of the microscope is 20 times.

To determine DNA concentration, the extracted nuclei were simultaneously stained with PI and 4′,6-diamidino-2-phenylindole (DAPI). The nuclei of the cells of healthy pulp showed no fluorescence (Figure 6A), while those of apples with bitter pit showed red fluorescence (Figure 6B). These results indicated that the nuclear membranes of the cells of healthy pulp remained intact, while the nuclear membranes of the pulp cells of apples with bitter pit lost selective permeability. The nuclei of cells of normal pulp and those of apples with bitter pit could be stained by DAPI, but the fluorescence density of healthy flesh cells was low and uniform (Figure 6A), while that of the pulp cells of apples with the bitter pit was high and uneven (Figure 6B). There was a greater number of high-intensity fluorescent spots, which indicated that the chromosomes in nuclei of the cells of fruits with bitter pit were highly concentrated. Similar to that in the animal cells, extensive chromatin condensation is the most conspicuous feature of plant cells undergoing PCD (Latrasse et al., 2016). The fluorescence of the nuclei, extracted from dexamethasone-treated flesh cells, stained with PI and DAPI was similar to that of the flesh cells of apples with bitter pit (Figure 6C). These findings demonstrated that the flesh cells of apples with bitter pit undergo PCD.

### Ultramicroscopic Observations of Amyloplasts in the Cells of Apples With Bitter Pit

**FIGURE 7:**
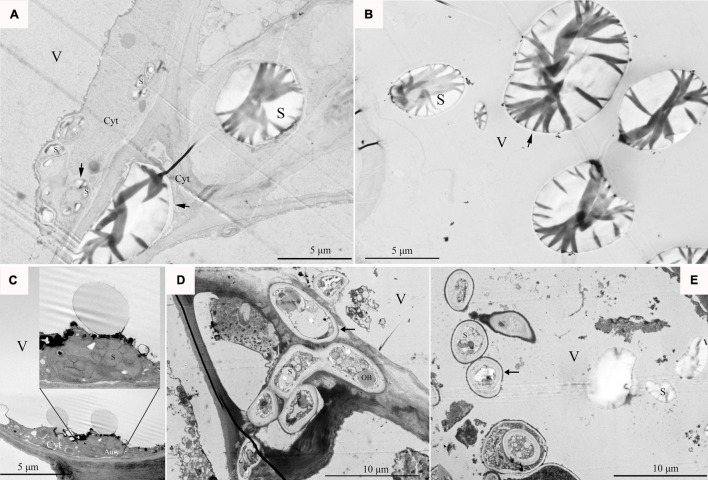
The state of amyloplasts in cells. **(A)** Flesh cell fragments of an apple with bitter pit, with a large amyloplast located in the cytoplasm. **(B)** Starch granules were freely lying in the vacuoles in the pulp cells of apples with bitter pit. **(C)** The amyloplasts contained starch granules and vesicles protruding into vacuoles. The inner image shows an enlarged amyloplast. **(D)** The amyloplast number increased *via* division, and the starch contained small starch granules and osmiophilic bodies. The red box shows amyloplast dividing in the protoplast and dissociating in the vacuole. **(E)** The amyloplasts were freely lying in the vacuoles. V, vacuoles; S, starch granule; OB, osmiophilic body. The arrow indicates the amyloplast similar to autophagy.

There were two kinds of amyloplasts in the pulp cells of apples with bitter pit. The first kind of amyloplasts was those containing large starch granules with an average size of 6.81 μm, (Supplementary Figure 5) some of which were located in the cytoplasm (Figure 7A) and some were lying freely in vacuoles (Figure 7B). The other kind contained small starch granules with an average size of 0.31 μm (Supplementary Figure 5). The amyloplasts containing small starch granules play a similar role as that of microautophagy (Figure 7C) in the programmed death of animal cells. This kind of amyloplasts can divide and proliferate (Figure 7D) and then move into the vacuole (Figure 7E). These results indicate that autophagy is involved in the apoptosis of flesh cells in apples with bitter pit.

## Discussion

The “Fuji” variety is one of the most common types of planted apples in the world, but it is susceptible to bitter pit (Bonomelli et al., 2020). A bitter pit can occur during both the fruit ripening and storage stages (Ferguson and Watkins, 1989), but we chose to study the fruit with a bitter pit in the ripening period. Although the storage temperature was approximately 0°C, the bitter pit spots of apples often developed a fungal infection, which affected the results of the experiment. It was relatively difficult to select an apple whose lesion spots were solely due to bitter pit during the storage stage. A bitter pit at the ripening stage of the fruit is characterized by the presence of sunken, water-soaked lesion spots (Supplementary Figures 1B,D) in the outer layers of the fruit. The cells of apples with bitter pit were observed under an optical microscope and were found to contain numerous amyloplasts; however, there were no amyloplasts in the healthy pulp cells, which were consistent with the results of a previous study (Simons and Chu, 1980). Blossom-end rot (BER) in pepper fruits is known as a physiological disorder, and the healthy part of BER fruits had higher concentrations of symplastic starch (Turhan et al., 2006). The amyloplasts are organelles found in plant cells that are responsible for starch synthesis and accumulation in plants. An amyloplast accumulation in the root cap occurs due to an increase in the cytoplasmic Ca^2+^ concentration (Chandra et al., 1982; Kolesnikov et al., 2016; Nakano et al., 2021). As assessed from the *A. thaliana* cell suspension, the amyloplasts are involved in a transient increase in Ca^2+^ concentration *via* several environmental stimuli, which suggests that the amyloplasts can balance Ca^2+^ concentrations (Sello et al., 2016) and that Ca^2+^ is closely related to the amyloplasts. The distribution of Ca^2+^ precipitated by potassium pyroantimonate in the cells of apples with the bitter pit was observed ultramicroscopically. It was found that a large amount of Ca^2+^ precipitated in the vacuoles and mitochondria. The area of Ca^2+^ precipitates represented the Ca^2+^ concentration (Otulak and Garbaczewska, 2011; Yin et al., 2015). In the fruit with bitter pit, the Ca^2+^ concentration in the cells in the bitter pit site was significantly higher than that in the cells close to the bitter pit site (Supplementary Figure 6). This finding indicates that there is a relationship between the amyloplasts and Ca^2+^ concentration in the pulp of apples with bitter pit.

Some studies have suggested that the bitter pit in fruits is caused by a decrease in free Ca^2+^ concentration in the apoplasts, which destroys the cell membrane and causes cell death (Miqueloto et al., 2014; DeBrouwer et al., 2020). Apoplastic water-soluble Ca^2+^ is closely related to physiological disorders, which are not affected by the season or harvest date. Pavicic et al. (2004) suggested that the deficiency of water-soluble Ca^2+^ is the most significant factor related to the development of bitter pit in “Idared” apples. However, according to our results, water-soluble calcium in the apoplast is not associated with the bitter pit. Furthermore, Liu et al. (2021) also found that the extracellular water-soluble Ca^2+^ in “Honeycrisp” apples is not associated with bitter pit. This is consistent with the results of the present study. We further investigated which kind of death of the cells of apples with bitter pit. In the present study, the flesh cells of apples with bitter pit were observed using Annexin V-FITC/PI staining. In addition, we used ultra-thin sections and cell nucleus extraction methods to determine the concentration of chromatin. The results were similar to those of oxidative stress-induced apoptosis in the diabetic islet cells (Srivastava et al., 2016) and suggested that the pulp cells of bitter pit undergo PCD. PCD is a physiological activity that regulates the body through active cell death (Hautegem et al., 2015). The process is carried out under the tight regulation of genes (Latrasse et al., 2016). Transcriptomic analysis revealed that the expression of genes that negatively regulate PCD in the flesh cells of apples with the bitter pit was higher than that in the healthy pulp cells (Supplementary Table 1). Furthermore, we transferred one of the different genes (*Md12g1174700*) to young tomato fruits using the transient method, which in turn induced PCD in tomatoes (Supplementary Figure 7). This further illustrated the PCD of the flesh cells of apples with bitter pit.

In animal cells, when the cell is programmed to die, the cell membrane can fold to form apoptotic bodies and phagosomes, but plant cells are surrounded by rigid cell walls and have no phagocytes; therefore, the system for degrading and recycling dying cells in plants is considered to be different to that in animals. In the starchy endosperm cells of rice or pericarp cells of wheat, the degradation of cell inclusions involves their digestion and absorption *via* the development of amyloplasts during PCD (Lan, 2004; Zhou et al., 2009). *Arabidopsis thaliana* genotypes grown under anthocyanin inductive conditions, and cotyledons can form autophagy containing anthocyanin deposits (Chanoca et al., 2015). Accordingly, we suggest that the amyloplasts formed in the pulp cells of apples with bitter pit also absorb degraded cell inclusions. In this case, the amyloplasts are involved in microautophagy. The amyloplasts contain small vesicles, starch granules, and an osmiophilic body (Supplementary Figure 8A). In *Arabidopsis* cotyledon cells, the microphages are formed when the tonoplast surrounds anthocyanin vacuolar inclusions and then enters the vacuole (Chanoca et al., 2015). However, in the flesh of apples with bitter pit, many cytoplasmic vesicles in the cells extend into the vacuole (Supplementary Figures 8B,C), which indicates that the amyloplasts enter the vacuoles by division.

In wheat endosperm cells, PCD can reduce the number of amyloids under waterlogged conditions (Fan et al., 1995; Herzog et al., 2016). During storage, the potato tubers degrade starch in the amyloplasts *via* PCD and destroy the amyloplast membranes (Sowokinos et al., 1987), but this type of degradation is not uniform across the tuber (Carvalho, 2017). During apple ripening, the starch in the cells of healthy fruits gradually transforms into soluble carbohydrates (Fan et al., 1995). Some fruits have low cytoplasmic Ca^2+^ concentrations, which leads to the destruction of amyloplast membranes, accelerates the degradation of starch, and increases the growth rate of the fruit and the concentration of soluble carbohydrates. This can also explain why the soluble carbohydrate content of the healthy pulp cells of fruits with the bitter pit was higher than that of healthy fruits, along with the weight of a single fruit, which was also higher than that of a healthy fruit (Supplementary Table 2). However, when soluble carbohydrate content increased, the number of amyloplasts increased. The amyloplasts may regulate cells to increase the Ca^2+^ concentration and maintain the integrity of the amyloplast membranes but disrupt Ca^2+^ distribution in fruit cells. In addition, the genes related to Ca^2+^ were highly expressed in the pulp of the fruit with bitter pit. Some studies have suggested that the reversal of the membrane damage in amyloplasts can help maintain their integrity and the accumulation of starch (Kumar and Knowles, 1993; Hepler, 2005).

The amyloplasts in bitter pit flesh cells can increase in number in a manner similar to that in rice endosperm cells. Moreover, when the number of amyloplast splits increases, the synthesis of starch also increases significantly (Kawagoe, 2009). PCD involves the degradation of starch in the amyloplasts. When the wheat suspension cell culture is cultured under sugar starvation, PCD is induced and begins to degrade the starch amyloplasts. However, the accumulation of starch in the pericarp cells of *Triticum aestivum* L. is accompanied by PCD. Interestingly, there are two contradictory processes in pericarp cells that occur simultaneously (Zhou et al., 2009). In this study, there was a large number of amyloplasts in the pulp cells of apples with bitter pit undergoing PCD at the same time. Ca^2+^ concentration increases in the early stage of post-harvest physiological deterioration of cassava storage roots, thereby causing PCD (Ren et al., 2021).

**FIGURE 8:**
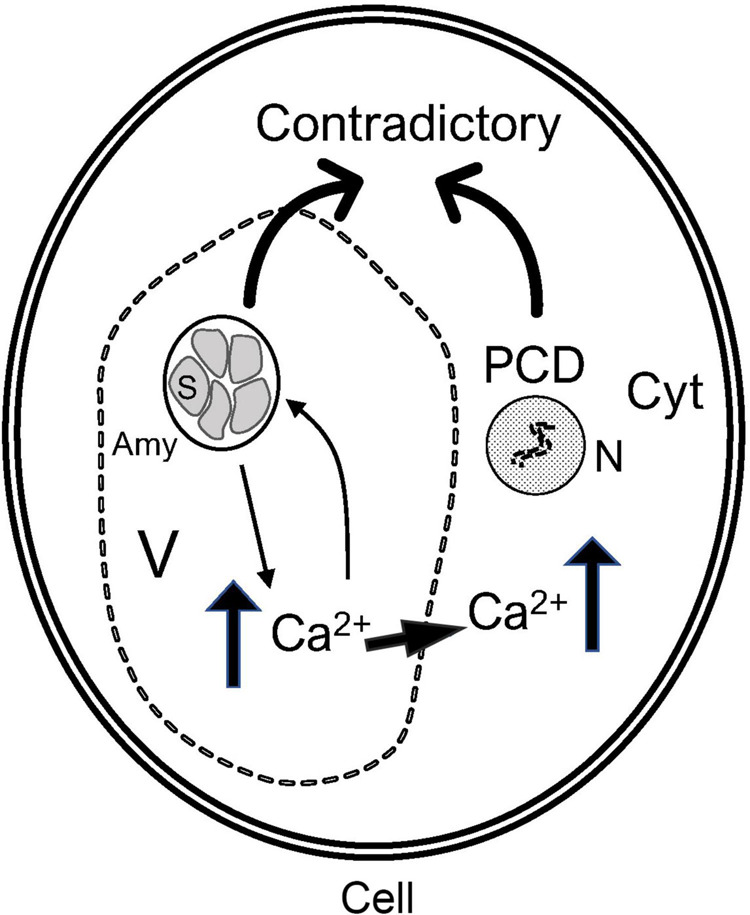
Patterns of interaction between the amyloplasts and Ca^2+^ inducing bitter pit in fruits. The low cytoplasmic Ca^2+^ concentration of fruits affects the degradation of amyloplasts, and amyloplasts disrupt the distribution of Ca^2+^ in cells, thereby causing metabolic disorders. Amy, amyloplast; S, starch granule; V, vacuole; Cyt, cytoplasm; N, nucleus.

In conclusion, the amyloplasts and Ca^2+^ regulate each other in pulp cells. Excessively large fruits were found to be highly susceptible to bitter pit. During fruit development, the concentration of Ca^2+^ in fruits showing rapid growth is lower (Saure, 2005). During fruit ripening, these types of fast-growing fruits can degrade the starch faster. Contrastingly, some pulp cells undergo the opposite process to that observed in amyloplasts, and the damaged amyloplasts can be restored. In cold-induced sweet potato, the integrity of amyloplasts can be restored because this process is reversible (Murphy et al., 2005). Intact amyloplasts can affect the distribution of Ca^2+^ in the fruit and increase the concentration of Ca^2+^ in cells. A high Ca^2+^ concentration could maintain the integrity of amyloplasts while inducing PCD. Thus, there are two contradictory processes in cells, the accumulation of amyloplasts and programmed death, that cause the metabolic disorder of the pulp cells, thereby leading to the formation of a bitter pit (Figure 8).

## Data Availability Statement

The datasets presented in this study can be found in online repositories. The names of the repository/repositories and accession number(s) can be found below: NCBI’s BioProject with accession number PRJNA733599 and SRA accession numbers SRR14684876, SRR14684877, and SRR14684878.

## Author Contributions

HQ and YW conceived and designed the experiments. LQ and SH performed the experiments. HQ analyzed the data. All authors contributed to the article and approved the submitted version.

## Conflict of Interest

The authors declare that the research was conducted in the absence of any commercial or financial relationships that could be construed as a potential conflict of interest.

## Publisher’s Note

All claims expressed in this article are solely those of the authors and do not necessarily represent those of their affiliated organizations, or those of the publisher, the editors and the reviewers. Any product that may be evaluated in this article, or claim that may be made by its manufacturer, is not guaranteed or endorsed by the publisher.

## Footnotes

1
http://www.biomarker.com.cn

2
www.biocloud.net

## Supplementary Material

Supplementary Figure 1 Comparison of healthy and bitter pit “Fuji” apples. **(A)** At maturity, fruit with a bitter pit on the tree, **(B)** healthy fruit, **(C)** fruit with bitter pit, **(D)** and the cut site of fruit with bitter pit. In general, there were no spots on the outer epidermis of the healthy fruits **(B)**, but fruits with bitter pit had several lesions in the outer peel **(C)**. Most of the lesions occurred near the calyx end and rarely occurred near the fruit stalk **(C)**. In the diseased part, the pulp under the peel developed brown lesions initially, and then, the symptoms spread to the outer skin. During the late stage of the disease, the flesh shrinks, and the epidermis becomes necrotic. The dark brown spots are 2–3 mm deep and the fruit has a bitter taste **(D)**.

Supplementary Figure 2 The free Ca^2+^ levels in pulp cells in apples with bitter pit were detected using the potassium pyroantimonate precipitation method. There was abundant Ca^2+^ granular precipitation on the vacuole membrane **(A,B)** and flocculent Ca^2+^ precipitation in the vacuole **(C,D)**. The white arrow indicates Ca^2+^ precipitation. Cw, cell wall; M, mitochondria; Cyt, cytoplasm; V, vacuole.

Supplementary Figure 3 Amyloplasts in the cells of apples with bitter pit. **(A)** Cells of the healthy pulp of the fruit with bitter pit. **(B)** Pulp cells near the site of the bitter pit. The red circle indicates an amyloplast. **(C)** Bitter pit pulp cells. The red arrow indicates an amyloplast.

Supplementary Figure 4 The expression of 6 genes was associated with Ca^2+^ concentration in pulp cells, as assessed *via* quantitative PCR. Control, healthy fruit; BP-H, healthy pulp of bitter pit fruit; BP, bitter pit fruit. Data are presented as mean ± SE. **Highly significant data (*P* < 0.01).

Supplementary Figure 5 We counted three bitter pit cells and measured six large starch granules and six small starch granules in each cell. Each starch granule was cross-measured two times, and the average value was taken as the size of a starch granule. Data are means ± SE. LSG, large starch granule; SSG, small starch granule.

Supplementary Figure 6 Comparison of the area of Ca^2+^ precipitation between the bitter pit pulp and the pulp close to the bitter pit spots. Three cells were randomly selected from a different region, and 10 Ca^2+^ precipitation particles were counted in each region. Data are presented as mean ± sSE (^∗∗∗^difference at *P* < 0.001). BP-C, the pulp close to a bitter pit spot; BP, bitter pit pulp.

Supplementary Figure 7 Through the transcriptomic analysis, five genes were found to be related to programmed cell death (PCD) (Supplementary Table 1). These genes were further confirmed *via* quantitative PCR. As *Md12g1174700* was the most differentially expressed gene (DEG) in the bitter pit and healthy tissues, it was transferred to young tomato fruits using the transient method. Seven days later, the tomatoes were picked, and the flesh was stained with Annexin V-FITC (red) and PI (green). The results of fluorescence observation suggested that the gene could induce PCD in tomatoes. **(A)** Cells of healthy tomato fruits, **(B)** cells of tomato fruits treated with dexamethasone, and **(C)** cells of tomato fruits after transient expression treatment. The arrow indicates the nucleus.

Supplementary Figure 8 Ultramicroscopic observation of amyloplasts and vesicles in the pulp cells of apples with bitter pit. **(A)** Amyloplasts are similar to autophagy. The amyloplasts contain small starch granules and osmiophilic bodies. The amyloid extends into the vacuole *via* division. The arrow indicates the amyloplast. S, starch granules; OB, osmiophilic bodies. **(B,C)** The protoplast had a large number of vesicles extending into the vacuole. The arrow indicates the vesicle.

Supplementary Table 1 The pulp tissue of healthy fruits, the flesh of the healthy part of the bitter pit fruit, and the flesh of the bitter pit-affected parts. RNA was extracted and transcriptomic analysis was performed. We identified five genes involved in PCD. The results showed that these genes were highly expressed in the bitter pit-affected parts of the fruits. This further illustrated the PCD of the flesh cells of apples with bitter pits.

Supplementary Table 2 The effect of the bitter pit on “Fuji” apple quality. Comparison of fruit solid content, single fruit weight, and fruit firmness between the bitter pit-affected fruit and healthy fruit. Three repetitions were performed with 10 fruits per repetition.
