# Supplementary material for: Accumulation of Abnormal Amyloplasts in Pulp Cells Induces Bitter Pit in Malus domestica
Source: Front Plant Sci. 2021 Sep 23;12:738726. doi: 10.3389/fpls.2021.738726 (PMC8496688; doi:10.3389/fpls.2021.738726)
Supplement: Supplementary Figure 1 — Comparison of healthy and bitter pit “Fuji” apples. (A) At maturity, fruit with a bitter pit on the tree, (B) healthy fruit, (C) fruit with bitter pit, (D) and the cut site of fruit with bitter pit. In general, there were no spots on the outer epidermis of the healthy fruits (B), but fruits with bitter pit had several lesions in the outer peel (C). Most of the lesions occurred near the calyx end and rarely occurred near the fruit stalk (C). In the diseased part, the pulp under the peel developed brown lesions initially, and then, the symptoms spread to the outer skin. During the late stage of the disease, the flesh shrinks, and the epidermis becomes necrotic. The dark brown spots are 2–3 mm deep and the fruit has a bitter taste (D). [file Presentation_1.PPTX]

## Slide 1
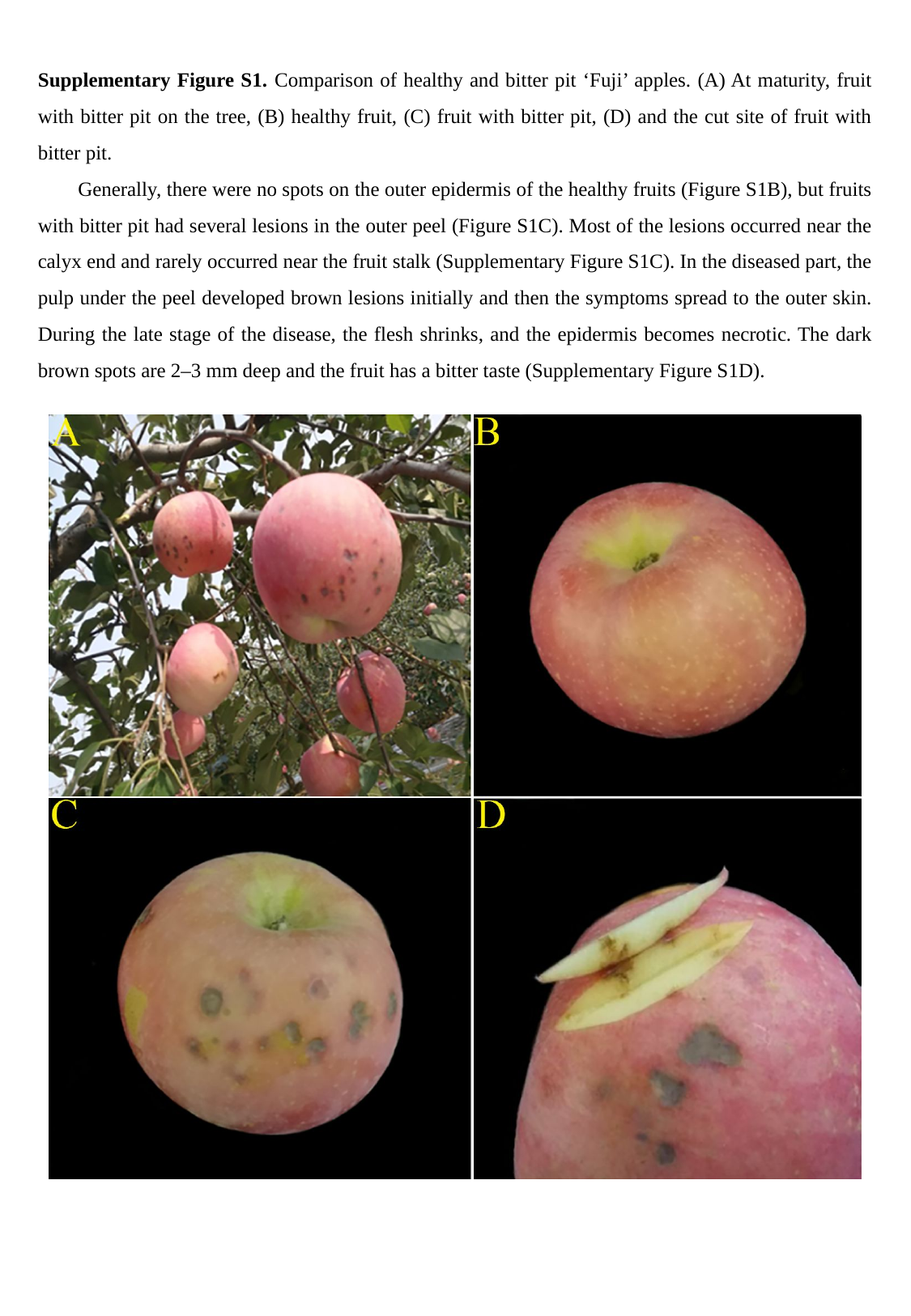

Supplementary Figure S1. Comparison of healthy and bitter pit ‘Fuji’ apples. (A) At maturity, fruit with bitter pit on the tree, (B) healthy fruit, (C) fruit with bitter pit, (D) and the cut site of fruit with bitter pit.
Generally, there were no spots on the outer epidermis of the healthy fruits (Figure S1B), but fruits with bitter pit had several lesions in the outer peel (Figure S1C). Most of the lesions occurred near the calyx end and rarely occurred near the fruit stalk (Supplementary Figure S1C). In the diseased part, the pulp under the peel developed brown lesions initially and then the symptoms spread to the outer skin. During the late stage of the disease, the flesh shrinks, and the epidermis becomes necrotic. The dark brown spots are 2–3 mm deep and the fruit has a bitter taste (Supplementary Figure S1D).
