# Supplementary material for: Accumulation of Abnormal Amyloplasts in Pulp Cells Induces Bitter Pit in Malus domestica
Source: Front Plant Sci. 2021 Sep 23;12:738726. doi: 10.3389/fpls.2021.738726 (PMC8496688; doi:10.3389/fpls.2021.738726)
Supplement: Supplementary Figure 2 — The free Ca2+ levels in pulp cells in apples with bitter pit were detected using the potassium pyroantimonate precipitation method. There was abundant Ca2+ granular precipitation on the vacuole membrane (A,B) and flocculent Ca2+ precipitation in the vacuole (C,D). The white arrow indicates Ca2+ precipitation. Cw, cell wall; M, mitochondria; Cyt, cytoplasm; V, vacuole. [file Presentation_2.PPTX]

## Slide 1
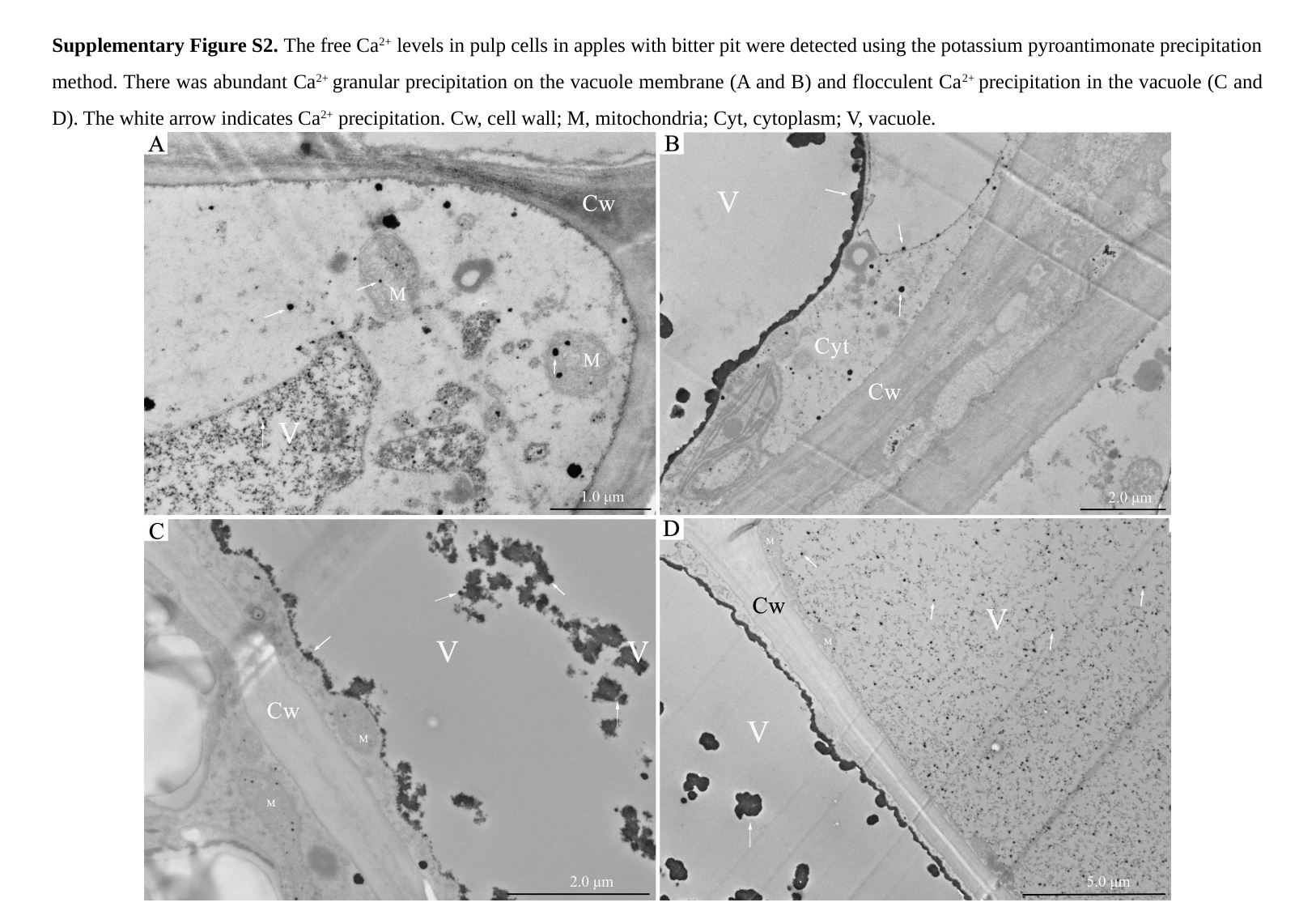

Supplementary Figure S2. The free Ca2+ levels in pulp cells in apples with bitter pit were detected using the potassium pyroantimonate precipitation method. There was abundant Ca2+ granular precipitation on the vacuole membrane (A and B) and flocculent Ca2+ precipitation in the vacuole (C and D). The white arrow indicates Ca2+ precipitation. Cw, cell wall; M, mitochondria; Cyt, cytoplasm; V, vacuole.
