# Supplementary material for: Accumulation of Abnormal Amyloplasts in Pulp Cells Induces Bitter Pit in Malus domestica
Source: Front Plant Sci. 2021 Sep 23;12:738726. doi: 10.3389/fpls.2021.738726 (PMC8496688; doi:10.3389/fpls.2021.738726)
Supplement: Supplementary Figure 3 — Amyloplasts in the cells of apples with bitter pit. (A) Cells of the healthy pulp of the fruit with bitter pit. (B) Pulp cells near the site of the bitter pit. The red circle indicates an amyloplast. (C) Bitter pit pulp cells. The red arrow indicates an amyloplast. [file Presentation_3.PPTX]

## Slide 1
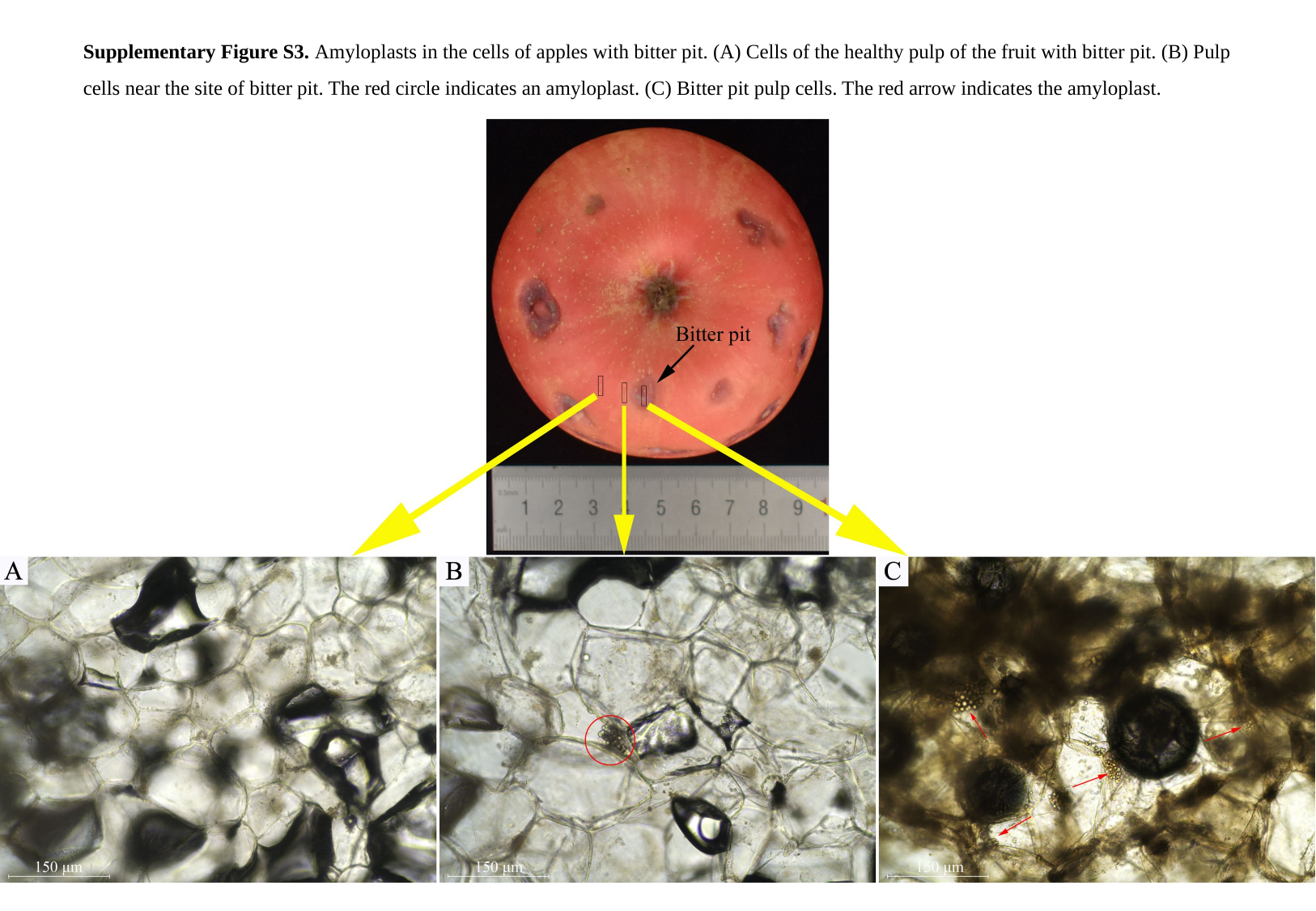

Supplementary Figure S3. Amyloplasts in the cells of apples with bitter pit. (A) Cells of the healthy pulp of the fruit with bitter pit. (B) Pulp cells near the site of bitter pit. The red circle indicates an amyloplast. (C) Bitter pit pulp cells. The red arrow indicates the amyloplast.
