# Supplementary material for: Accumulation of Abnormal Amyloplasts in Pulp Cells Induces Bitter Pit in Malus domestica
Source: Front Plant Sci. 2021 Sep 23;12:738726. doi: 10.3389/fpls.2021.738726 (PMC8496688; doi:10.3389/fpls.2021.738726)
Supplement: Supplementary Figure 4 — The expression of 6 genes was associated with Ca2+ concentration in pulp cells, as assessed via quantitative PCR. Control, healthy fruit; BP-H, healthy pulp of bitter pit fruit; BP, bitter pit fruit. Data are presented as mean ± SE. **Highly significant data (P < 0.01). [file Presentation_4.PPTX]

## Slide 1
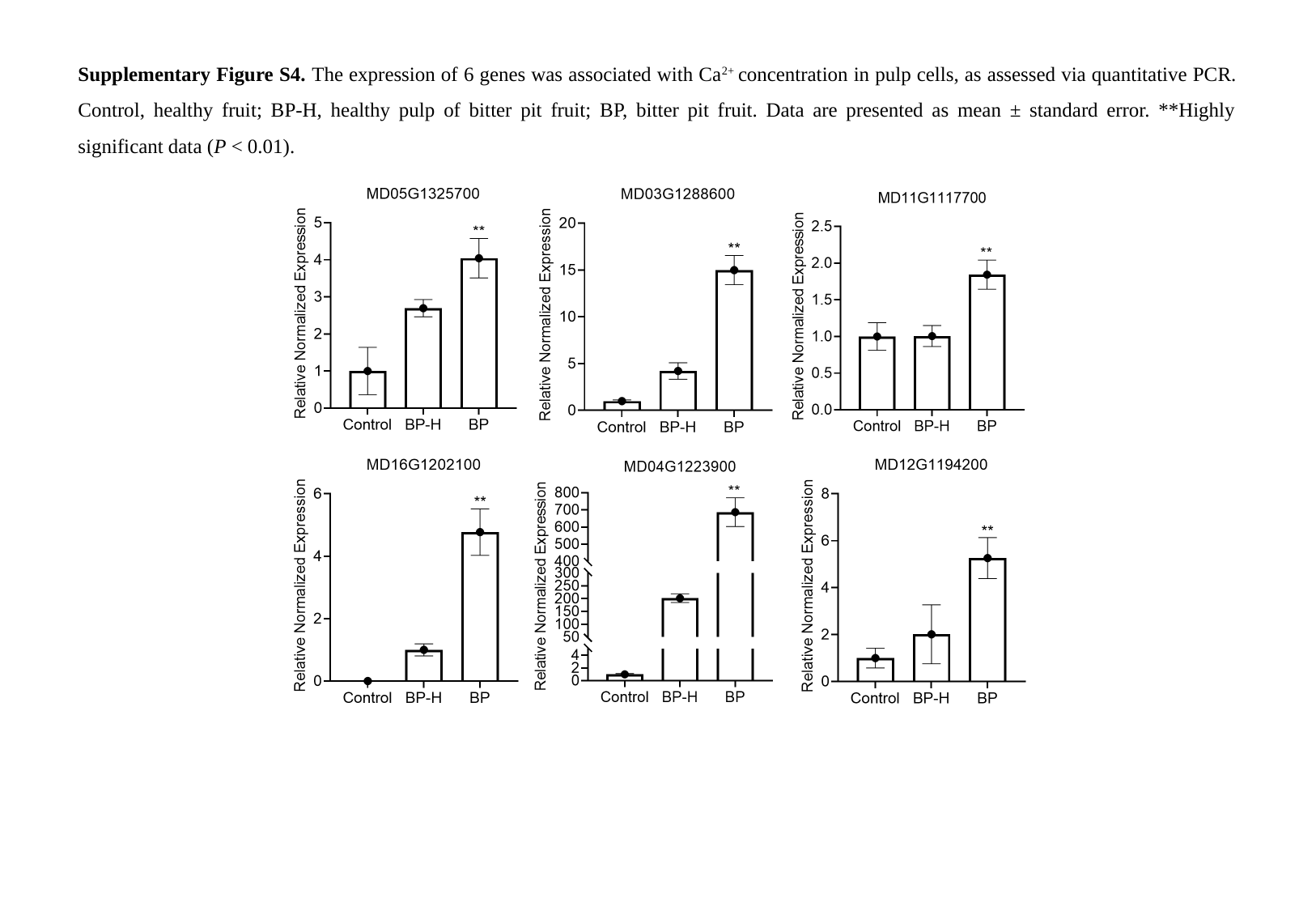

Supplementary Figure S4. The expression of 6 genes was associated with Ca2+ concentration in pulp cells, as assessed via quantitative PCR. Control, healthy fruit; BP-H, healthy pulp of bitter pit fruit; BP, bitter pit fruit. Data are presented as mean ± standard error. **Highly significant data (P < 0.01).
