# Supplementary material for: Accumulation of Abnormal Amyloplasts in Pulp Cells Induces Bitter Pit in Malus domestica
Source: Front Plant Sci. 2021 Sep 23;12:738726. doi: 10.3389/fpls.2021.738726 (PMC8496688; doi:10.3389/fpls.2021.738726)
Supplement: Supplementary Figure 5 — We counted three bitter pit cells and measured six large starch granules and six small starch granules in each cell. Each starch granule was cross-measured two times, and the average value was taken as the size of a starch granule. Data are means ± SE. LSG, large starch granule; SSG, small starch granule. [file Presentation_5.PPTX]

## Slide 1
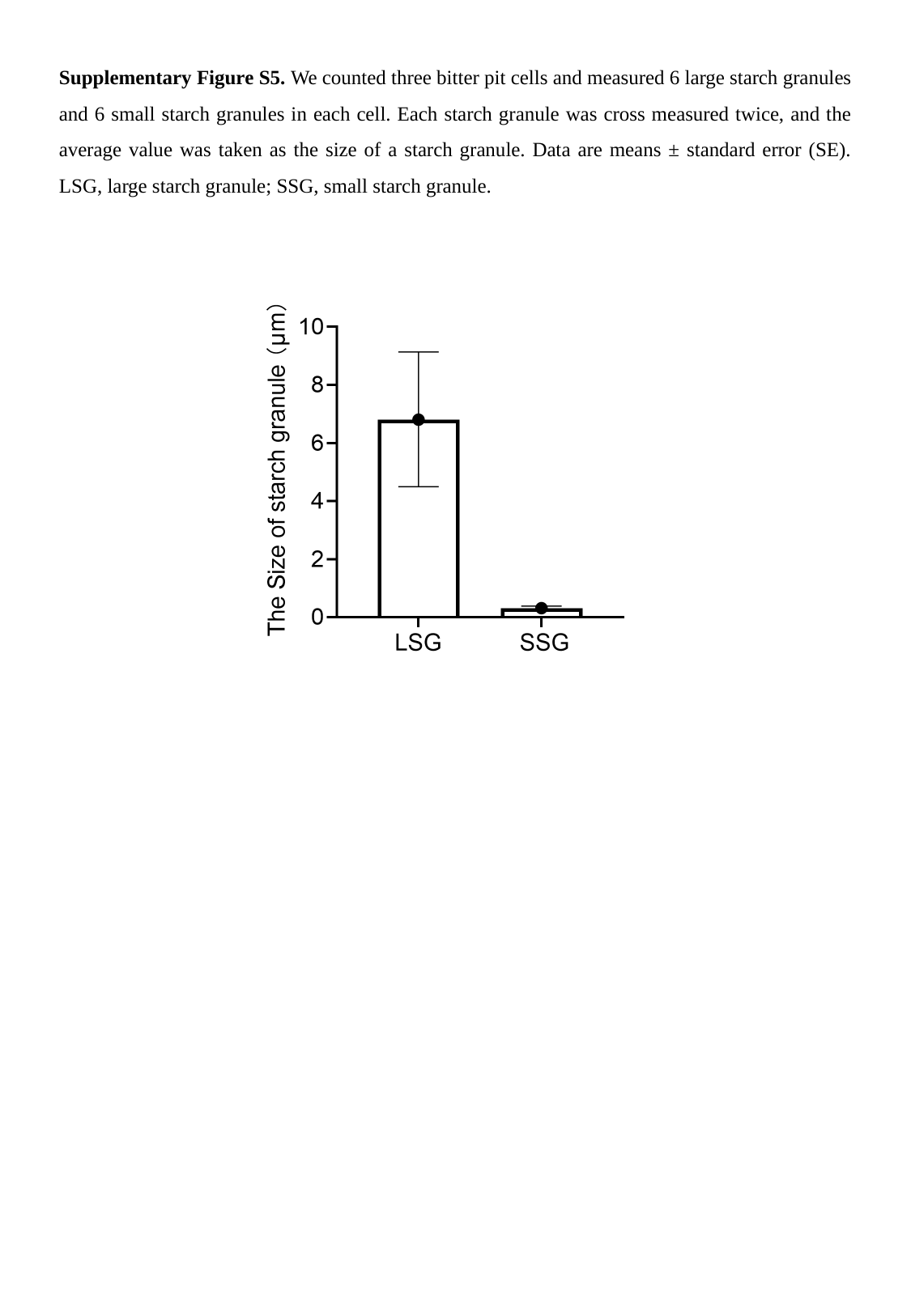

Supplementary Figure S5. We counted three bitter pit cells and measured 6 large starch granules and 6 small starch granules in each cell. Each starch granule was cross measured twice, and the average value was taken as the size of a starch granule. Data are means ± standard error (SE). LSG, large starch granule; SSG, small starch granule.
