# Supplementary material for: Accumulation of Abnormal Amyloplasts in Pulp Cells Induces Bitter Pit in Malus domestica
Source: Front Plant Sci. 2021 Sep 23;12:738726. doi: 10.3389/fpls.2021.738726 (PMC8496688; doi:10.3389/fpls.2021.738726)
Supplement: Supplementary Figure 6 — Comparison of the area of Ca2+ precipitation between the bitter pit pulp and the pulp close to the bitter pit spots. Three cells were randomly selected from a different region, and 10 Ca2+ precipitation particles were counted in each region. Data are presented as mean ± sSE (∗∗∗difference at P < 0.001). BP-C, the pulp close to a bitter pit spot; BP, bitter pit pulp. [file Presentation_6.PPTX]

## Slide 1
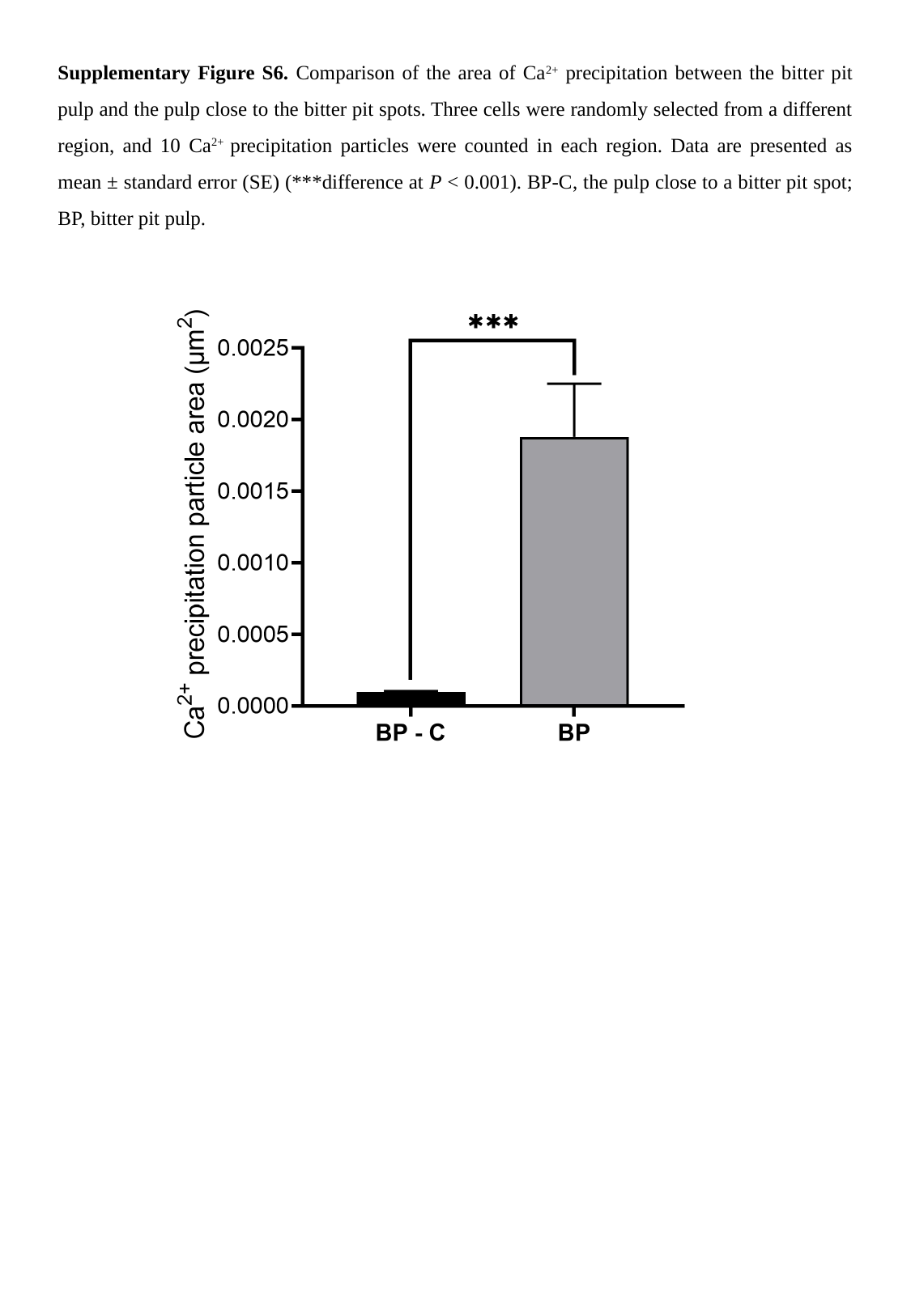

Supplementary Figure S6. Comparison of the area of Ca2+ precipitation between the bitter pit pulp and the pulp close to the bitter pit spots. Three cells were randomly selected from a different region, and 10 Ca2+ precipitation particles were counted in each region. Data are presented as mean ± standard error (SE) (***difference at P < 0.001). BP-C, the pulp close to a bitter pit spot; BP, bitter pit pulp.
