# Supplementary material for: Accumulation of Abnormal Amyloplasts in Pulp Cells Induces Bitter Pit in Malus domestica
Source: Front Plant Sci. 2021 Sep 23;12:738726. doi: 10.3389/fpls.2021.738726 (PMC8496688; doi:10.3389/fpls.2021.738726)
Supplement: Supplementary Figure 7 — Through the transcriptomic analysis, five genes were found to be related to programmed cell death (PCD) (Supplementary Table 1). These genes were further confirmed via quantitative PCR. As Md12g1174700 was the most differentially expressed gene (DEG) in the bitter pit and healthy tissues, it was transferred to young tomato fruits using the transient method. Seven days later, the tomatoes were picked, and the flesh was stained with Annexin V-FITC (red) and PI (green). The results of fluorescence observation suggested that the gene could induce PCD in tomatoes. (A) Cells of healthy tomato fruits, (B) cells of tomato fruits treated with dexamethasone, and (C) cells of tomato fruits after transient expression treatment. The arrow indicates the nucleus. [file Presentation_7.PPTX]

## Slide 1
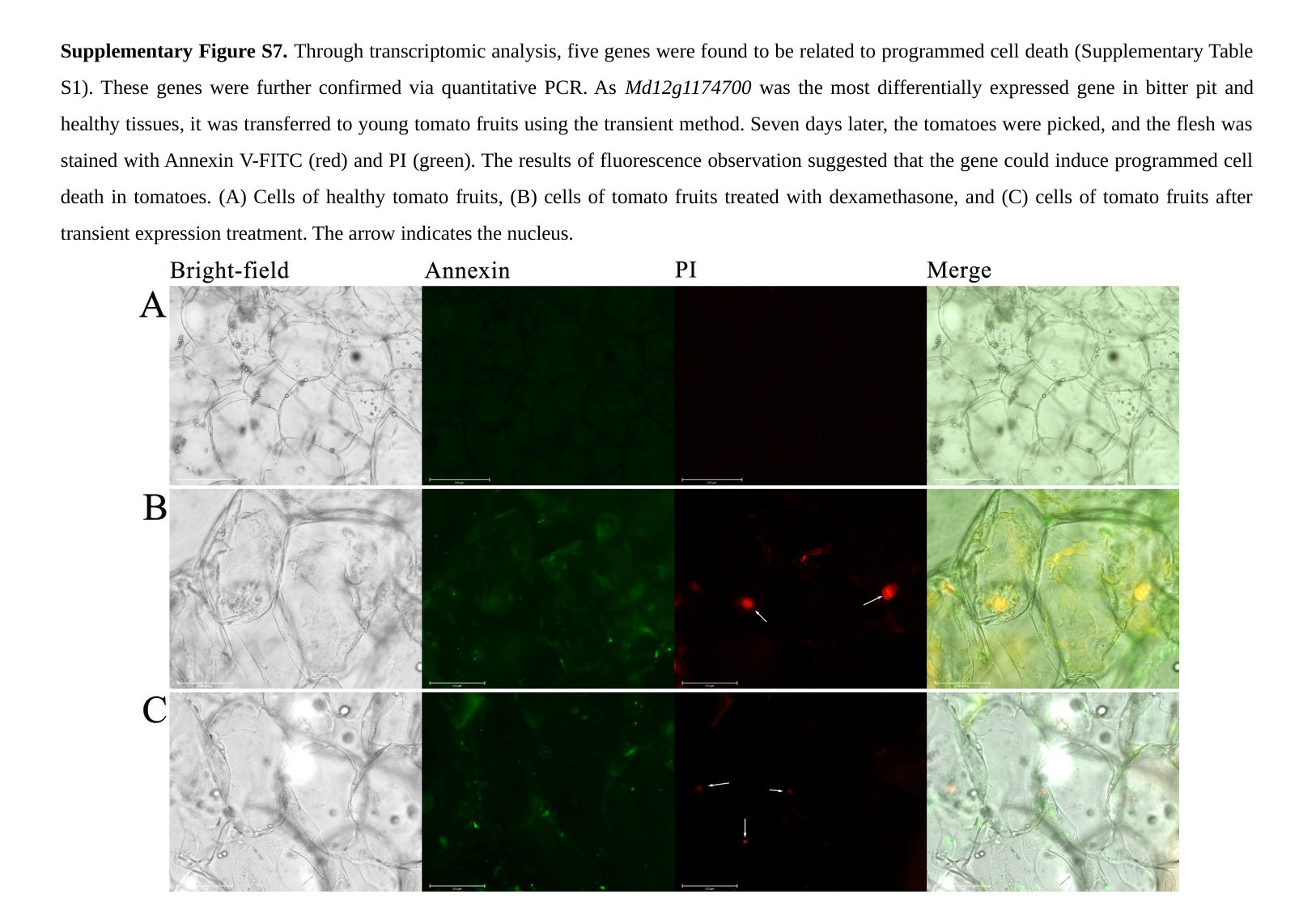

Supplementary Figure S7. Through transcriptomic analysis, five genes were found to be related to programmed cell death (Supplementary Table S1). These genes were further confirmed via quantitative PCR. As Md12g1174700 was the most differentially expressed gene in bitter pit and healthy tissues, it was transferred to young tomato fruits using the transient method. Seven days later, the tomatoes were picked, and the flesh was stained with Annexin V-FITC (red) and PI (green). The results of fluorescence observation suggested that the gene could induce programmed cell death in tomatoes. (A) Cells of healthy tomato fruits, (B) cells of tomato fruits treated with dexamethasone, and (C) cells of tomato fruits after transient expression treatment. The arrow indicates the nucleus.
