# Supplementary material for: Accumulation of Abnormal Amyloplasts in Pulp Cells Induces Bitter Pit in Malus domestica
Source: Front Plant Sci. 2021 Sep 23;12:738726. doi: 10.3389/fpls.2021.738726 (PMC8496688; doi:10.3389/fpls.2021.738726)
Supplement: Supplementary Figure 8 — Ultramicroscopic observation of amyloplasts and vesicles in the pulp cells of apples with bitter pit. (A) Amyloplasts are similar to autophagy. The amyloplasts contain small starch granules and osmiophilic bodies. The amyloid extends into the vacuole via division. The arrow indicates the amyloplast. S, starch granules; OB, osmiophilic bodies. (B,C) The protoplast had a large number of vesicles extending into the vacuole. The arrow indicates the vesicle. [file Presentation_8.PPTX]

## Slide 1
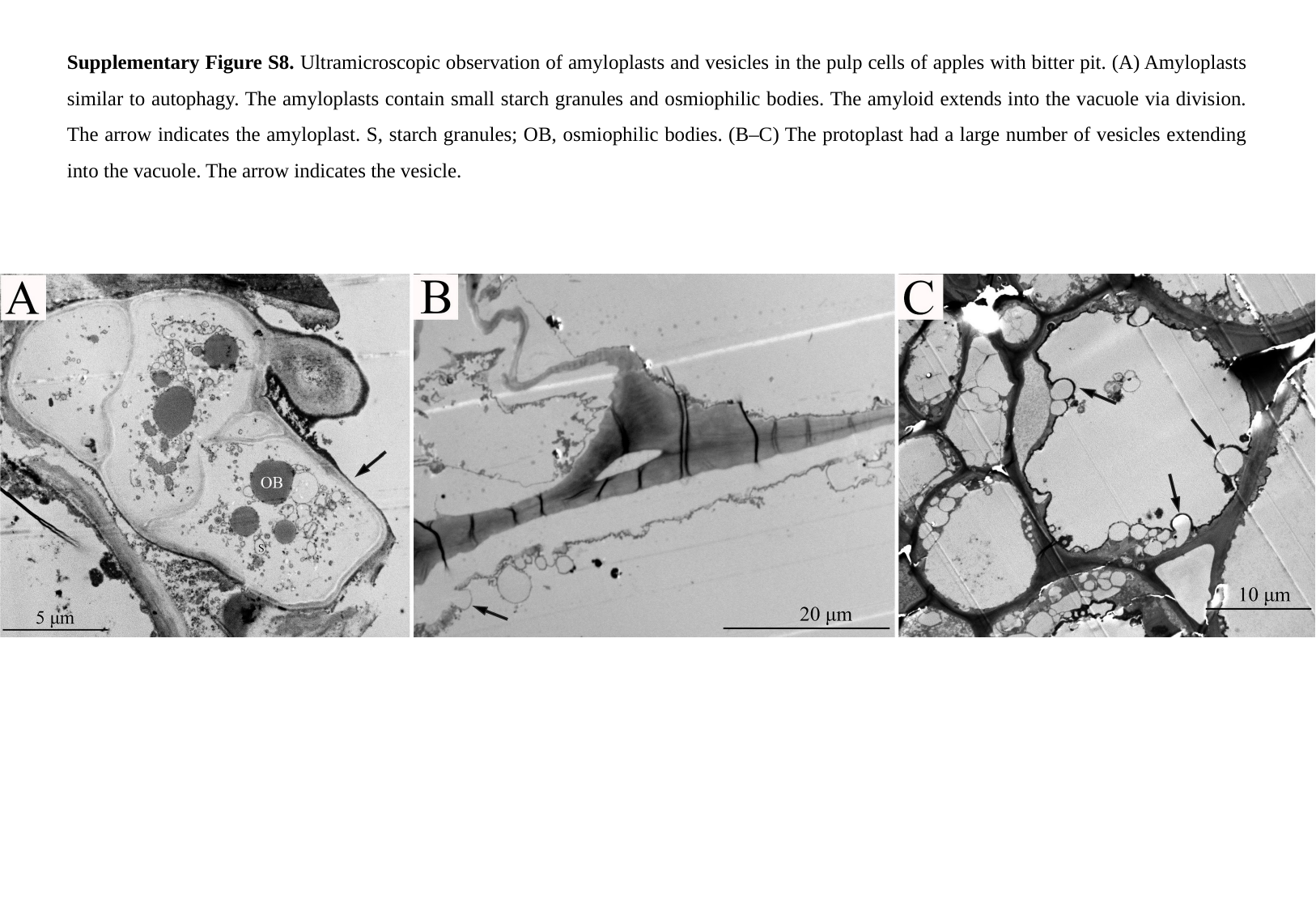

Supplementary Figure S8. Ultramicroscopic observation of amyloplasts and vesicles in the pulp cells of apples with bitter pit. (A) Amyloplasts similar to autophagy. The amyloplasts contain small starch granules and osmiophilic bodies. The amyloid extends into the vacuole via division. The arrow indicates the amyloplast. S, starch granules; OB, osmiophilic bodies. (B–C) The protoplast had a large number of vesicles extending into the vacuole. The arrow indicates the vesicle.
