# Supplementary material for: Accumulation of Abnormal Amyloplasts in Pulp Cells Induces Bitter Pit in Malus domestica
Source: Front Plant Sci. 2021 Sep 23;12:738726. doi: 10.3389/fpls.2021.738726 (PMC8496688; doi:10.3389/fpls.2021.738726)
Supplement: Supplementary Table 1 — The pulp tissue of healthy fruits, the flesh of the healthy part of the bitter pit fruit, and the flesh of the bitter pit-affected parts. RNA was extracted and transcriptomic analysis was performed. We identified five genes involved in PCD. The results showed that these genes were highly expressed in the bitter pit-affected parts of the fruits. This further illustrated the PCD of the flesh cells of apples with bitter pits. [file Table_1.DOC]

**Supplementary Table 1.** The pulp tissue of healthy fruits, the flesh of the healthy part of the bitter pit fruit, and the flesh of the bitter pit-affected parts. RNA was extracted and transcriptomic analysis was performed. We identified five genes involved in programmed cell death. The results showed that these genes were highly expressed in the bitter pit-affected parts of the fruits. This further illustrated the programmed cell death of the flesh cells of apples with bitter pits.

| GeneID | Log_2_^(T01/T02)^ | Log_2_^(T01/T03)^ | GO_annotation | NR_annotation |
| --- | --- | --- | --- | --- |
| MD12G1174700 | 1.92 | 6.50 | Biological Process: negative regulation of programmed cell death (GO:0043069) | PREDICTED: ammonium transporter 2-like [*Malus domestica*] |
| MD13G1017300 | 1.69 | 5.13 | Biological Process: negative regulation of programmed cell death (GO:0043069) | PREDICTED: U-box domain-containing protein 21-like [*Malus domestica*] |
| MD16G1015400 | 1.77 | 6.33 | Biological Process: negative regulation of programmed cell death (GO:0043069) | PREDICTED: U-box domain-containing protein 21-like [*Malus domestica*] |
| MD09G1111800 | 1.79 | 5.51 | Biological Process: regulation of programmed cell death (GO:0043067 | PREDICTED: lysM domain receptor-like kinase 3 isoform X1 [*Pyrus x bretschneideri*] |
| MD04G1162200 | 1.57 | 5.67 | Biological Process: negative regulation of programmed cell death (GO:0043069) | PREDICTED: ammonium transporter 2-like, partial [*Malus domestica*] |

Note:

(1) T01 represents the flesh of apples with bitter pit. T02 represents the healthy part of apples with bitter pit. T03 represents the flesh of healthy apples.

(2) Positive values represent up-regulated gene and Negative values represent the down regulated genes. FDR-corrected P ≤ 0.0005
