# Supplementary material for: Accumulation of Abnormal Amyloplasts in Pulp Cells Induces Bitter Pit in Malus domestica
Source: Front Plant Sci. 2021 Sep 23;12:738726. doi: 10.3389/fpls.2021.738726 (PMC8496688; doi:10.3389/fpls.2021.738726)
Supplement: Supplementary Table 2 — The effect of the bitter pit on “Fuji” apple quality. Comparison of fruit solid content, single fruit weight, and fruit firmness between the bitter pit-affected fruit and healthy fruit. Three repetitions were performed with 10 fruits per repetition. [file Table_2.DOCX]

**Supplementary Table S2.** The effect of bitter pit on ‘Fuji’ apple quality. Comparison of fruit solid content, single fruit weight, and fruit firmness between the bitter pit-affected fruit and healthy fruit. Three repetitions were performed with 10 fruits per repetition.

| Fruit types | Single fruit weight  (g) | Soluble solid  (%) | Fruit firmness (kg/cm^2^) |
| --- | --- | --- | --- |
| Bitter pit | 432.62 ± 9.77 a | 13.51 ± 0.38 a | 9.37 ± 0.19 a |
| Healthy fruit (Control) | 353.68 ± 11.41 b | 12.10 ± 0.23 b | 9.73 ± 0.16 a |

Note: Different small letters mean significant difference (P<0.05) in the same column. Data are means ± standard error (SE)
